# CCN2 Enhances Resistance to Cisplatin-Mediating Cell Apoptosis in Human Osteosarcoma

**DOI:** 10.1371/journal.pone.0090159

**Published:** 2014-03-17

**Authors:** Hsiao-Chi Tsai, Chun-Yin Huang, Hong-Lin Su, Chih-Hsin Tang

**Affiliations:** 1 Department of Life Sciences, National Chung Hsing University, Taichung, Taiwan; 2 Department of Orthopaedic Surgery, China Medical University Beigang Hospital, Yun-Lin County, Taiwan; 3 Graduate Institute of Clinical Medical Science, China Medical University, Taichung, Taiwan; 4 Graduate Institute of Basic Medical Science, China Medical University, Taichung, Taiwan; 5 Department of Pharmacology, School of Medicine, China Medical University, Taichung, Taiwan; 6 Department of Biotechnology, College of Health Science, Asia University, Taichung, Taiwan; Boston University Goldman School of Dental Medicine, United States of America

## Abstract

Osteosarcoma (OS) is the most common form of malignant bone tumor and is an aggressive malignant neoplasm exhibiting osteoblastic differentiation. Cisplatin is one of the most efficacious antitumor drugs for osteosarcoma patients. However, treatment failures are common due to the development of chemoresistance. CCN2 (also known as CTGF), is a secreted protein that binds to integrins, modulates the invasive behavior of certain human cancer cells. However, the effect of CCN2 in cisplatin-mediated chemotherapy is still unknown. Here, we found that CCN2 was upregulated in human osteosarcoma cells after treatment with cisplatin. Moreover, overexpression of CCN2 increased the resistance to cisplatin-mediated cell apoptosis. In contrast, reduction of CCN2 by CCN2 shRNA promoted the chemotherapeutic effect of cisplatin. We also found that CCN2 provided resistance to cisplatin-induced apoptosis through upregulation of Bcl-xL and survivin. Knockdown of Bcl-xL or survivin removed the CCN2-mediated resistance to apoptosis induced by cisplatin. On the other hand, CCN2 also promoted FAK, MEK, and ERK survival signaling pathways to enhance tumor survival during cisplatin treatment. In a mouse xenograft model, overexpression of CCN2 promoted resistance to cisplatin. However, knockdown of CCN2 increased the therapeutic effect of cisplatin. Therefore, our data suggest that CCN2 might be a critical oncogene of human osteosarcoma for cisplatin-resistance and supported osteosarcoma cell growth *in vivo* and *in vitro*.

## Introduction

Osteosarcoma is the most common form of malignant bone tumor and is also the eighth most common form of childhood cancer. In pediatric patients, approximately 20% of all primary bone cancers constitute 2.4% of all malignancies. Osteosarcoma arises from mesenchymal cells and is pathologically characterized by spindle cells and aberrant osteoid formation. The incidence of osteosarcoma has a bimodal distribution in adolescence and in the seventh and eighth decade of life. Cisplatin is one of the most effective drugs against osteosarcoma [1]. However, in the past few years, increasing chemoresistance has led to a decrease in the number of osteosarcoma patients who improve [2]. Thus, the analysis of the molecular mechanisms involved in the chemoresistance of osteosarcoma cells is pivotal to improving patient survival.

There are 6 CCN family members, including CCN1 (cysteine-rich protein 61, Cyr61), CCN2 (connective tissue growth factor, CTGF), CCN3 (nephroblastoma overexpressed gene, Nov), CCN4 (Wnt-1-induced secreted protein 1, WISP-1), CCN5 (WISP-2), and CCN6 (WISP-3) [3]. The biological properties of CCN proteins include stimulation of cellular migration, adhesion, proliferation, extracellular matrix (ECM) formation, and regulation of tumorigenesis [4]. Many studies have confirmed that CCN2 has a higher level of expression in lung cancer [5], pancreatic cancer [6], breast cancer [7], chondrosarcoma [8], and melanomas [9] than in normal tissues. Overexpression of CCN2 in tumor cells has also been linked to increased tumor size and lymph node metastasis [10]. Previous studies have demonstrated that CCN2 expression confers resistance to chemotherapeutic agents in glioblastoma [11] and ovarian cancer [12]. In chondrosarcoma patients, the expression of CCN2 also correlates with the patient survival ratio [13]. Therefore, these data suggest that CCN2 expression may be involved in the progression and chemoresistance of human cancers.

CCN2 interacts with integrin to regulate a number of biological functions [14]. It is commonly believed that survival of adherent cells require signal transduction from the interactions between integrins and the extracellular matrix (ECM). Such signals are in turn transmitted to the cytoplasm by components of the focal adhesions, in which focal adhesion kinase (FAK) is a considerable player. The major site of FAK autophosphorylation, Tyr^397^, is important for the biochemical and biological functions of FAK [15]. On the other hand, FAK-dependent MEK/ERK activation regulates expression of many functional genes that affect cell survival, proliferation, and differentiation [16]. ERK1/2 also has profound effects on the regulation of apoptosis by regulating the phosphorylation of apoptotic regulatory molecules, including Bad, Bim, Mcl-1, caspase-9, and Bcl-2 [17]. The hyperactivation of ERK has been shown to promote resistance to chemotherapy drugs in many cancer cells [17], [18]. Furthermore, survivin is a member of the inhibitor of apoptosis (IAP) family. Strong survivin expression is observed in the vast majority of cancers [19]. The survivin protein can inhibit cell apoptosis or programmed cell death through inhibiting functional caspase activation [20]. However, the signaling pathways in CCN2-mediated chemoresistance in human osteosarcoma are mostly unclear and largely unknown.

Although the role of CCN2 in chemoresistance has been implicated in some cancer cells, the effect of CCN2 on cisplatin-induced cell apoptosis in human osteosarcoma has not been extensively studied. In this study, we found that CCN2 enhanced resistance to cisplatin-increasing cell death in human osteosarcoma *in vivo* and *in vitro*. Our results provide evidence that CCN2 might be a novel chemotherapy target in human osteosarcoma cells.

## Materials and Methods

### Materials

Anti-rabbit and anti-mouse IgG-conjugated horseradish peroxidase, rabbit polyclonal antibodies (specific for FAK, p-MEK, MEK, p-ERK, and ERK), mouse monoclonal antibodies (specific for CCN2, Bcl-xL, surviving, poly[ADP-ribose] polymerase [PARP], and α-tubulin), and small interfering RNAs (siRNAs) against Bcl-xL, survivin, and control (for experiments using targeted siRNA transfection; each consists of a scrambled sequence that will not lead to the specific degradation of any known cellular mRNA) were purchased from Santa Cruz Biotechnology (Santa Cruz, CA, USA). Rabbit polyclonal antibody specific for p-FAK was purchased from Cell Signaling and Neuroscience (Danvers, MA, USA). MEK inhibitors (PD98059 and U0126) were purchased from Calbiochem (San Diego, CA, USA). The phosphorylation site mutant of FAK^(Y397F)^ was a gift from Dr. J. A. Girault (Institut du Fer à Moulin, Moulin, France). The MEK1 dominant-negative mutant was provided by Dr. W. M. Fu (National Taiwan University, Taipei, Taiwan). The ERK2 ^(K52R)^ dominant-negative mutant was a gift from Dr. M. Cobb (University of Texax, Dallas, TX). All other chemicals were purchased from Sigma-Aldrich (St. Louis, MO, USA).

### Cell culture

The human osteosarcoma cell lines (MG-63, U-2 OS, and HOS), human lung adenocarcinoma cell lines (A549), human prostate cancer cell lines (PC3), and human gastric adenocarcinoma epithelial cell line (AGS) were purchased from the American Type Cell Culture Collection (Manassas, VA, USA). MG-63 and HOS cells were maintained in Eagle's Minimum Essential Medium. A549 and AGS cells were maintained in Nutrient Mixture Ham's F12 medium. PC3 cells were maintained in Roswell Park Memorial Institute (RPMI) 1640 medium. All the mediums were supplemented with 20 mM HEPES and 10% heat-inactivated FCS, 2 mM glutamine, penicillin (100 U/ml), and streptomycin (100 µg/ml) at 37°C with 5% CO_2_. U-2 OS cells were maintained in McCoy's 5A medium, which was supplemented with 10% FBS, penicillin (100 U/ml), and streptomycin (100 µg/ml) at 37°C with 5% CO_2_.

### Overexpression of CCN2 with the pcDNA3.1- CCN2 expression vector

The complete CCN2 open reading frame was amplified by reverse transcription (RT)-PCR. Subsequent PCR amplification from RT reaction products was performed in 0.2 mM dNTPs, 1.5 mM MgCl2, 40 U/ml of Platinum® *Pfx* DNA Polymerase (Invitrogen, Groningen, The Netherlands), and 1 nmol of each PCR primer, designed to amplify the full-length CCN2 cDNA (sense: CCAACCATGACCGCCGCCAG, and antisense: TCATGCCATGTCTCCGTACATCTTCCTG). PCR products were purified from agarose gels using the Viogene Gel/PCR DNA Isolation System (Viogene, CA, USA). The complete CCN2 was cloned into the topoisomerase-activated pcDNA3.1-TOPO vector (Invitrogen).

### 3-(4,5-dimethylthiazol-2-yl)-2,5-diphenyltetrazolium bromide (MTT) assay

Cell viability was determined by the MTT assay. Cells were plated in 96-well plates at a concentration of 2,000 cells per well. After treatment with cisplatin or doxorubicin for 24 h, cultures were washed with PBS [21]. Next, 0.5 mg/ml of MTT solution was added to each well and incubated at 37°C for 30 min. To dissolve formazan crystals, culture medium was replaced with an equal volume of DMSO. After the mixture was shaken at room temperature for 10 min, absorbance of each well was determined at 550 nm using a microplate reader (Bio-Tek, Winooski, VT, USA).

### Colony formation assay

Cells were plated in 12-well plates at a concentration of 1×10^4^ cells per well. After treatment with cisplatin for 48 h, cells were washed with PBS and replaced with fresh medium. Cells were allowed to form colonies for 7 days before being stained with crystal violet (0.4 mg/ml). After washing 3 times with PBS, acetic acid was added to a final concentration of 33% (v/v), and the absorbance was measured at 550 nm.

### Quantification of apoptosis by flow cytometry

Quantitative assessment of apoptotic cells was assessed by examining the cell cycle. Cells were collected by centrifugation and adjusted to 3×10^6^ cells/ml. Pre-chilled ethanol was added to 0.5-ml cell suspensions, and the mixture was incubated at 4°C for 30 min. Ethanol was removed by centrifugation, and cellular DNA was stained with 100 µg/ml propidium iodide (PI; in PBS containing 0.1% Triton-X 100, and 1 mM EDTA) in the presence of an equal volume of DNase-free RNase (200 µg/ml). After staining, cells were analyzed immediately with a FACScan and Cellquest program. The extent of apoptosis was determined by measuring the DNA content of cells below the sub-G1 peak.

A terminal deoxynucleotidyl transferase-mediated deoxyuridine triphosphate nick end labeling (TUNEL) assay was also used to examine cell apoptosis using the BD ApoAlert™ DNA Fragmentation Assay Kit (BD Biosciences, California, USA). Cells were incubated with cisplatin for 24 h, trypsinized, fixed with 4% paraformaldehyde, and permeabilized with 0.1% Triton-X-100 in 0.1% sodium citrate. After being washed with PBS, the cells were incubated with the reaction mixture for 60 min at 37°C. The stained cells were then analyzed with a flow cytometer.

### 4′-6-diamidino-2-phenylindole (DAPI) staining

Apoptotic nuclei were detected using DAPI staining. Cells were plated in 6-well plates at a concentration of 1×10^6^ cells per well. After being treated with cisplatin at various concentrations for 48 h, cells were washed with PBS, fixed with 4% paraformaldehyde, and analyzed via fluorescence microscopy to assess chromatin condensation and segregation.

### Caspase-3 activity assay

Caspase-3 activity was measured by the direct assay of caspase-3 enzyme activity in cell lysates using synthetic chromogenic substrate (Ac-DEVD-pNA; substrate for caspase-3). Cell lysates were prepared and incubated with anti-caspase-3. Immunocomplexes were incubated with peptide substrate in assay buffer (100 mM NaCl, 50 mM 4-(2-hydroxyethyl)-1-piperazine-ethanesulphonic acid [HEPES], 10 mM dithiothreitol, 1 mM EDTA, 10% glycerol, and 0.1% 3-[(3-cholamidopropyl) dimethylammonio]-1-propanesulfonate [CHAPS], pH 7.4) for 2 h at 37°C. The release of p-nitroaniline was monitored at 405 nm. Results are the percent change in activity compared to an untreated control.

### Western blotting analysis

The cellular lysates were prepared as described previously [22]. Protein concentration was determined using the Thermo Scientific Pierce BCA Protein Assay Kit (Thermo Fisher Scientific Inc., Waltham, MA). Proteins were resolved on SDS-PAGE and transferred to immobilon polyvinyldifluoride (PVDF) membranes. The blots were blocked with 4% BSA for 1 h at room temperature and incubated with the following primary antibodies for 1 h at room temperature to detect antigen: mouse monoclonal anti-CCN2, Bcl-xL, survivin, or α-tubulin (Santa Cruz Biotechnology) or rabbit polyclonal anti-FAK, p-MEK, MEK, p-ERK, or ERK (Santa Cruz Biotechnology). After 3 washes in tris-buffered saline with 0.05% Tween 20 (TBS-Tween), the blots were subsequently incubated with a donkey anti-rabbit or anti-mouse peroxidase-conjugated secondary antibody for 1 h at room temperature. The blots were visualized by enhanced chemiluminescence using Kodak X-OMAT LS film (Eastman Kodak, Rochester, NY). Quantitative data were obtained using a computing densitometer and ImageQuant software (Molecular Dynamics, Sunnyvale, CA).

### Quantitative real-time PCR

Total RNA was extracted from osteosarcoma cells using a TRIzol kit (MDBio Inc., Taipei, Taiwan). The reverse transcription reaction was performed using 2 µg of total RNA that was reverse transcribed into cDNA using an oligo(dT) primer. A volume of 100 ng total cDNA was added per 25-µl reaction, along with sequence-specific primers and Taqman® probes. Sequences for all target gene primers and probes were purchased commercially (β-actin was used as the internal control) (Applied Biosystems, CA). qPCR assays were carried out in triplicate using a StepOnePlus sequence detection system. The cycling conditions were 10 min of polymerase activation at 95°C, followed by 40 cycles at 95°C for 15 s and 60°C for 60 s. The threshold was set above the non-template control background and within the linear phase of target gene amplification to calculate the cycle number at which the transcript was detected (denoted as CT).

### Mouse xenograft models

To generate murine subcutaneous tumors, 1×10^6^ osteosarcoma cells were injected subcutaneously to the right flanks of the nude mice (purchased from the National Science Council Animal Center, Taipei, Taiwan). Four weeks after injection, the subcutaneous tumor size had reached a tumor volume of approximately 100 mm^3^, and the mice then received intraperitoneal (i.p.) injections of cisplatin (15 mg/kg) twice a week thereafter. Tumor volumes were calculated by the following formula: length×width^2^×π/6. This study was carried out in strict accordance with the recommendations in the Animal Care and Use Guidelines of the China Medical University (Taichung, Taiwan). The protocol was approved by the Affidavit of Approval of Animal Use Protocol China Medical University (Permit Number: 100-27-N). All surgery was performed under Tricholoroacetaldehyde Monohydrate, and all efforts were made to minimize suffering.

### Statistics

The values given are mean ± SEM. The significance of difference between the experimental groups and controls was assessed by Student's *t* test. The difference was considered significant if the *p* value was less than 0.05.

## Results

### Cisplatin increases CCN2 expression in osteosarcoma cells

**Figure 1:**
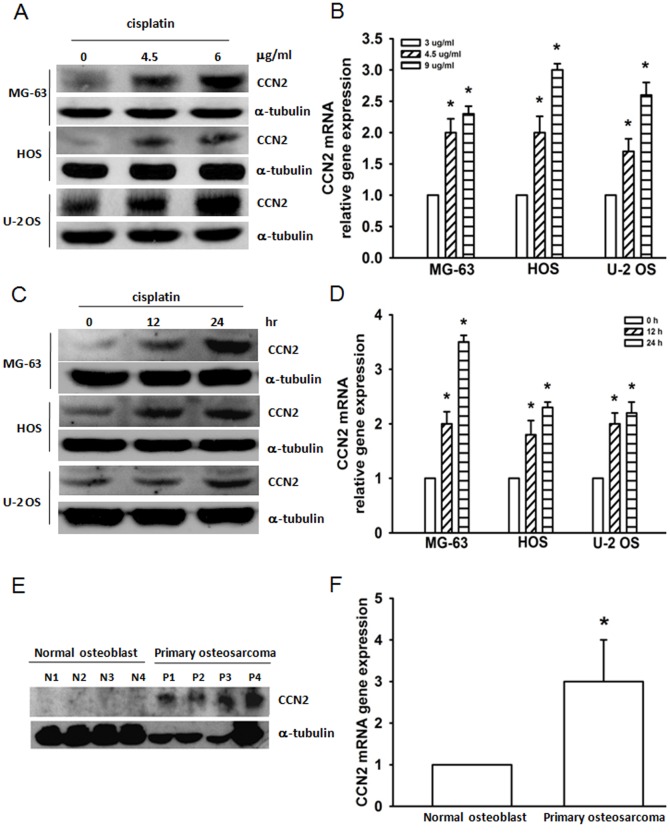
The expression of CCN2 in human osteosarcoma. Cells were treated with cisplatin in different time or dose intervals, and the protein and mRNA expression of CCN2 was examined by western blotting (A & C) and qPCR (B & D). (E & F) The protein and mRNA expression of CCN2 in primary osteoblast cells and osteosarcoma cells were examined by western blotting and qPCR. Results are expressed as mean ± SEM. *, p<0.05 as compared with control group.

Previous evidence has shown that in human squamous lung carcinoma, 42 genes showed increases or decreases in expression of more than 2-fold with cisplatin treatment. CCN2 is 1 of the 5 genes that showed the highest degree of variations in its expressions [23]. Therefore, we hypothesized that CCN2 may be involved in chemotherapy of cisplatin in human osteosarcoma cells. First, we treated osteosarcoma cell lines (MG-63, HOS, and U-2 OS) with cisplatin and examined CCN2 expression. Incubation of osteosarcoma cell lines with cisplatin significantly increased CCN2 protein and mRNA expressions in dose and time dependent manner (Fig. 1A–D). In addition, we also found that the protein and mRNA expression of CCN2 in primary osteosarcoma was significantly higher than in normal osteoblasts (Fig. 1E&F). These findings suggested that CCN2 is upregulated during chemotherapy in osteosarcoma cells.

### Overexpression of CCN2 enhances resistance to cisplatin-promoting cell death

**Figure 2:**
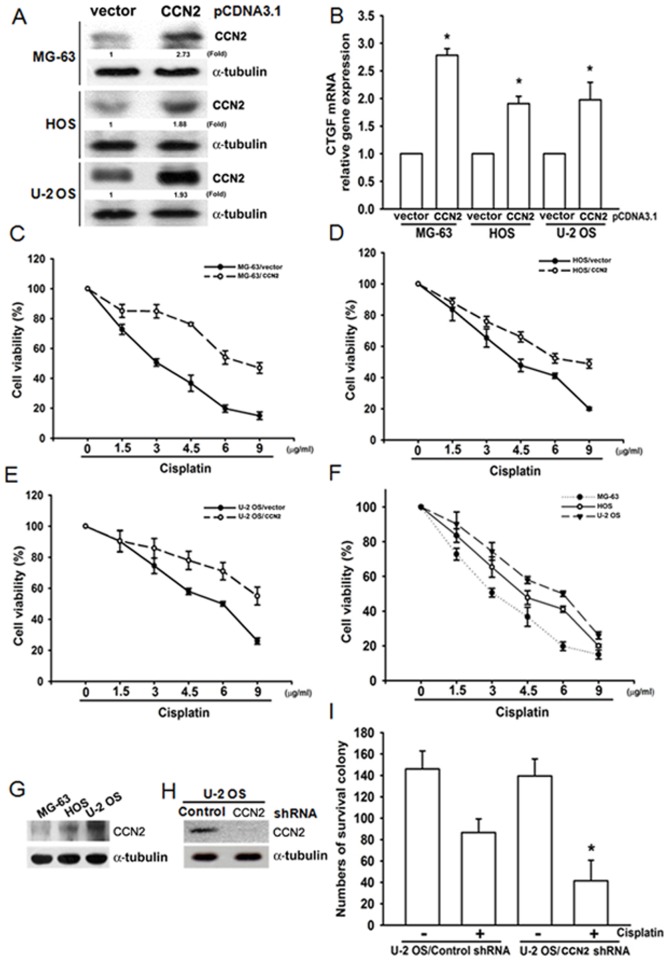
Overexpression of CCN2 enhances resistance to cisplatin-mediated cell death. (A & B) CCN2-overexpressing osteosarcoma cells were established using pCDNA3.1-CCN2 vector, and protein and mRNA expression of CCN2 were examined by western blotting and qPCR. (C–F) Cells were treated with cisplatin for 24 h, and cell viability was analyzed by MTT assay. (G) The CCN2 expression in MG-63, HOS, and U-2 OS cells was detected by western blotting. (H) U-2 OS cells were transfected with control or CCN2 shRNA, and CCN2 expression was examined by western blotting. (I) Cells were treated with cisplatin (4.5 µg/ml) for 24 h and then provided with fresh medium. After 1 week in culture, the colonies were stained by crystal violet. Each experiment was done in triplicate. Results are expressed as mean ± SEM. *, p<0.05 as compared with control group.

To examine the potential role of CCN2 on the regulation of chemoresistance to cisplatin, we established vector expressing control cells (MG-63/vector, HOS/vector, and U-2 OS/vector cells) and overexpressing CCN2 cells (MG-63/CCN2, HOS/CCN2, and U-2 OS/CCN2 cells). As shown in Figure 2A and 2B, MG-63/CCN2, HOS/CCN2, and U-2 OS/CCN2 cells expressed higher protein and mRNA levels of CCN2 than in MG-63/vector, HOS/vector, and U-2 OS/vector cells. Previous studies have demonstrated that CCN2 expression confers resistance to chemotherapeutic agents in glioblastoma [11] and ovarian cancer [12]. Therefore, we hypothesized that CCN2 may promote resistance to cisplatin in human osteosarcoma cells. Using the MTT assay, we found that overexpression of CCN2 protected cisplatin-induced cell death (Figs. 2C–E). However, overexpression of CCN2 did not affect basal cell proliferation in human osteosarcoma cells (Figure S1). Doxorubicin is another chemotherapeutic agent for human osteosarcoma treatment [24]. We next examine whether CCN2 protected doxorubicin-mediated cell death. The results also showed that CCN2 protected doxorubicin-induced cell death in human osteosarcoma cells (Figure S2). We next compared chemoresistance after cisplatin stimulation between 3 osteosarcoma cell lines. We found that U-2 OS cells were more resistant than MG63 and HOS cells (Fig. 2F). In addition, western blotting revealed a higher level of expression of CCN2 in U-2 OS cells and a lower level in MG63 cells (Fig. 2G). Therefore, CCN2 expression is associated with a chemoresistant phenotype of osteosarcoma cells. To examine whether inhibition of CCN2 reduced resistance to cisplatin, we created stable U-2 OS cell lines expressing CCN2 short hairpin RNA (U-2 OS/CCN2 shRNA) and control short hairpin RNA (U-2 OS/Control shRNA). Western blotting results confirmed that stable U-2 OS/CCN2 shRNA cells significantly decreased the protein expression of CCN2 (Fig. 2H). Using a colony formation assay, we found that knockdown of CCN2 expression in U-2 OS cells drastically decreased clonogenic ability upon exposure to cisplatin (Fig. 2I). These data suggest that CCN2 plays an important role in increasing the resistance of osteosarcoma cells to cisplatin.

### CCN2-induced resistance is mediated through inhibition of apoptosis

**Figure 3:**
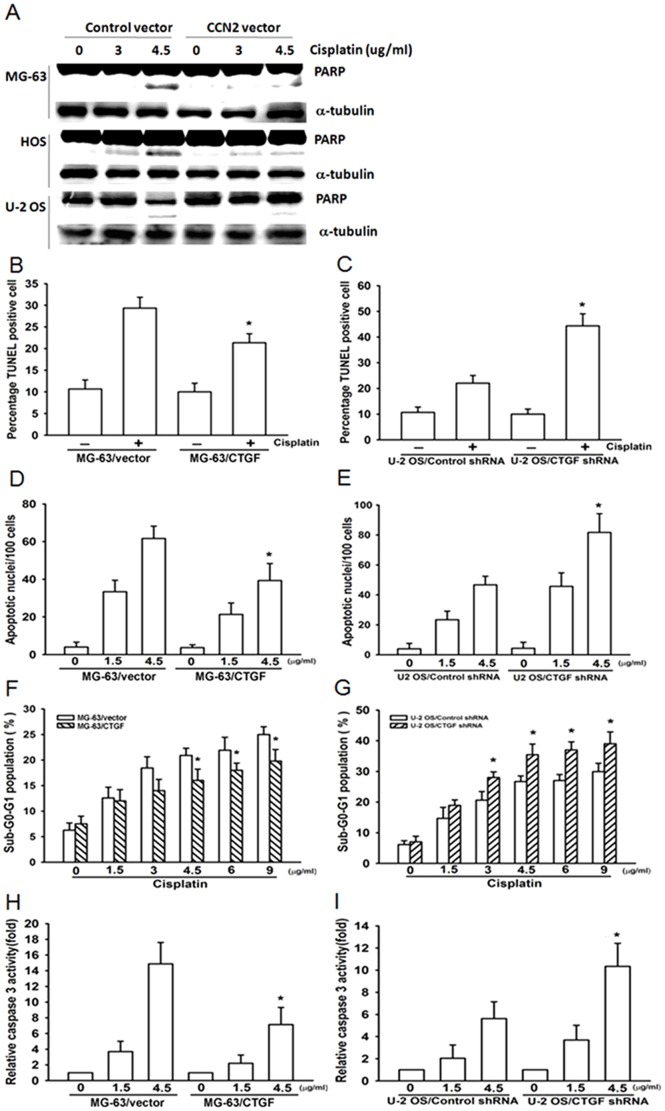
CCN2-induced resistance is mediated through inhibition of apoptosis. (A) Cells were treated with cisplatin for indicated concentration for 24 h, and PARP expression was examined by western blotting. (B–G) Cells were treated with cisplatin (4.5 µg/ml) for 24 h, and cell apoptosis was examined by TUNEL analysis (B & C), DAPI assay (D & E), PI staining (F & G), and caspase-3 activity (H & I). Each experiment was done in triplicate. Results are expressed as mean ± SEM. *, p<0.05 as compared with control group.

Cancer therapy failure is often related to decreased sensitivity to apoptosis in resistant tumors [25]. PARP cleavage serves as a marker for cells undergoing apoptosis [26]. We examined PARP expression to determine whether CCN2-induced resistance is mediated through inhibition of apoptosis. As shown in Figure 3A, cisplatin treatment caused strong PARP cleavage in MG-63/vector, HOS/vector, and U-2 OS/vector cells when compared with cells overexpressing CCN2. To further confirm that CCN2-induced resistance is mediated through inhibition of apoptosis, osteosarcoma cells were incubated with cisplatin, and cell apoptosis was examined by TUNEL staining, DAPI staining, PI staining, and caspase-3 activity assays. We found that overexpression of CCN2 significantly decreased cisplatin-mediated cell apoptosis (Fig. 3B, D, F, and H). In contrast, decreased CCN2 expression promoted cisplatin-induced apoptosis of cells in osteosarcoma (Figs. 3C, E, G, and I). These data confirmed that CCN2-induced resistance is mediated through inhibition of apoptosis.

### Bcl-xL and survivin are involved in CCN2-mediated chemoresistance

**Figure 4:**
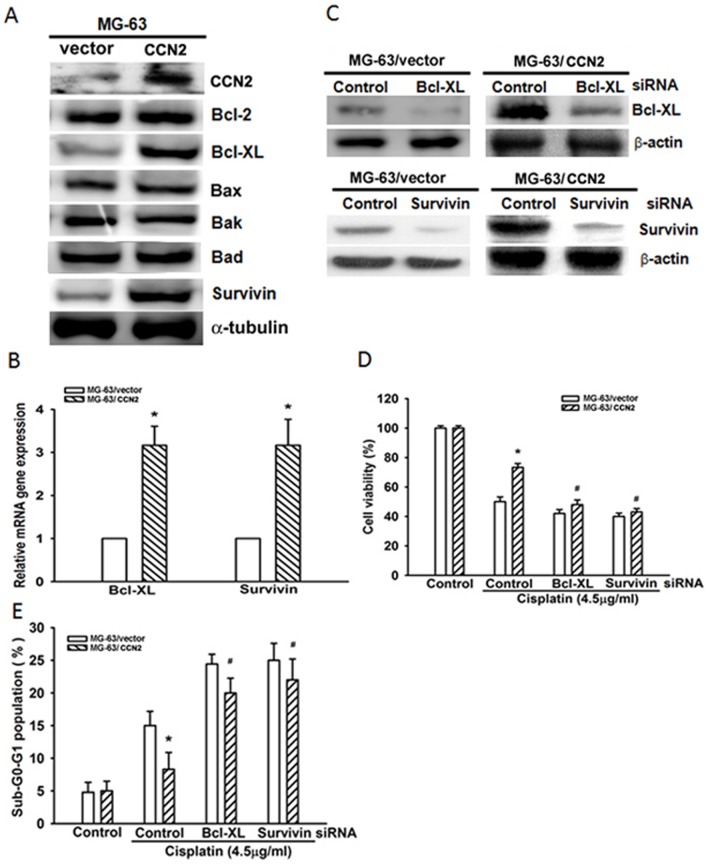
Bcl-xL and survivin are involved in CCN2-mediated chemoresistance. (A) Protein expression of the Bcl-2 family was examined by western blotting. (B) Cells were treated with cisplatin (4.5 µg/ml) for 24 h, and the mRNA expression of Bcl-xL and survivin was examined by qPCR. (C) Cells were transfected with control, Bcl-xL, or survivin siRNA, and the protein expression was examined by western blotting. (D & E) Cells were transfected with control, Bcl-xL, or survivin siRNA, and cell viability and apoptosis was analyzed by MTT assay and PI staining. Each experiment was done in triplicate. Results are expressed as mean ± SEM. *, p<0.05 as compared with MG-63/vector group; ^#^ *P*<0.05 compared with MG-63/CCN2 cisplatin-treated control group.

Activation of the mitochondrial apoptosis pathway plays a central role in cisplatin-induced cell death [27]. To investigate whether mitochondrial signaling is involved in CCN2-mediated resistance, protein expression of the Bcl-2 family was examined. We found that overexpression of CCN2 increased Bcl-xL but not Bcl-2, Bax, Bak, or Bad expression (Figs. 4A & B). Survivin expression has been reported to be an indicator of poor prognosis, low apoptotic index, poor differentiation, and high proliferation index and might be a promising option in the treatment of osteosarcoma [28], [29], [30], [31]. Overexpression of CCN2 also increased survivin expression in human osteosarcoma (Figs. 4A & B). To further confirm whether upregulation of Bcl-xL and survivin by CCN2 participate in the resistance to cisplatin-induced cell apoptosis, we assessed the effects of siRNAs targeting Bcl-xL and survivin. Protein expression of Bcl-xL or survivin was effectively downregulated by transfection with Bcl-xL or survivin siRNA, respectively (Fig. 4C). On the other hand, transfection of cells with Bcl-xL or survivin siRNA reversed CCN2-mediated chemoresistance to cisplatin-induced cell death (Figs. 4D & E). According to these results, Bcl-xL and survivin are important downstream effectors in CCN2-enhanced resistance from cisplatin-mediated cell apoptosis in osteosarcoma cells.

### CCN2 activates FAK, MEK, and ERK survival signaling pathways to subsequently protect cisplatin-induced cell apoptosis

**Figure 5:**
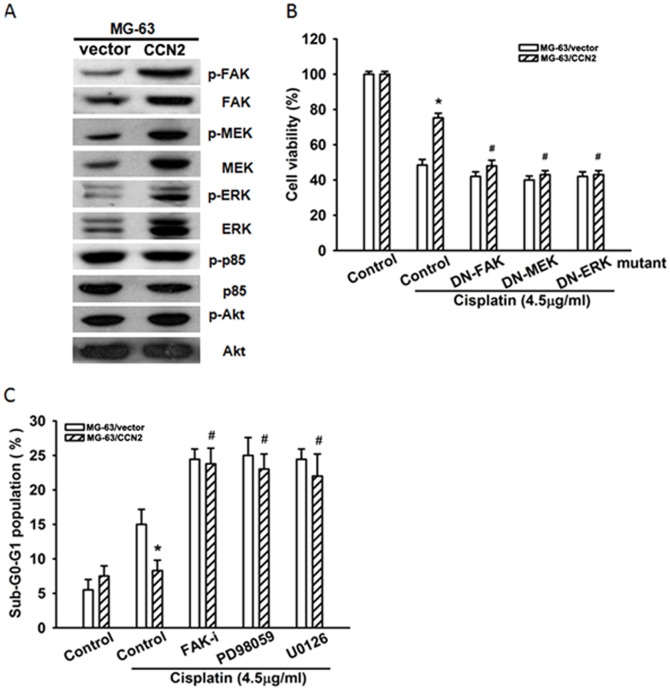
CCN2 activates FAK, MEK, and ERK survival signaling pathways during chemoresistance. (A) Protein expression was examined by western blotting. (B & C) Cells were transfected with FAK, MEK, and ERK mutants or pretreated with FAK inhibitor (10 µM), PD98059 (10 mM), and U0126 (10 µM), followed by stimulation with cisplatin for 24 h. Cell survival ability and apoptosis were examined by MTT assay and PI staining. Each experiment was done in triplicate. Results are expressed as mean ± SEM. *, p<0.05 as compared with MG-63/vector group; ^#^ *P*<0.05 compared with MG-63/CCN2 cisplatin-treated control group.

Resistance to cancer therapy not only decreases sensitivity to apoptosis but also alternative pathways to promote cell survival [25]. FAK-dependent MEK/ERK activation is a common survival signaling pathway [16]. We found that FAK, MEK, and ERK phosphorylation increased in MG-63-overexpressing CCN2 cells (Fig. 5A). On the other hand, overexpression of CCN2 did not activate other survival signaling molecules, such as PI3K and Akt (Fig. 5A). We next examined whether CCN2-mediated FAK, MEK, and ERK activation promoted cell survival during cisplatin treatment. Transfection of cells with FAK, MEK, and ERK mutants or pretreatment of cells with FAK inhibitor and MEK inhibitors (PD98059 or U0126) diminished the CCN2-mediated chemoresistance (Figs. 5B & C). However, these inhibitors did not affect cell viability in osteosarcoma cells (data not shown). Therefore, CCN2 also activated FAK, MEK, and ERK survival pathways, subsequently conveying resistance to cisplatin-induced cell apoptosis.

### CCN2 confers drug resistance to cisplatin in a mouse xenograft model

**Figure 6:**
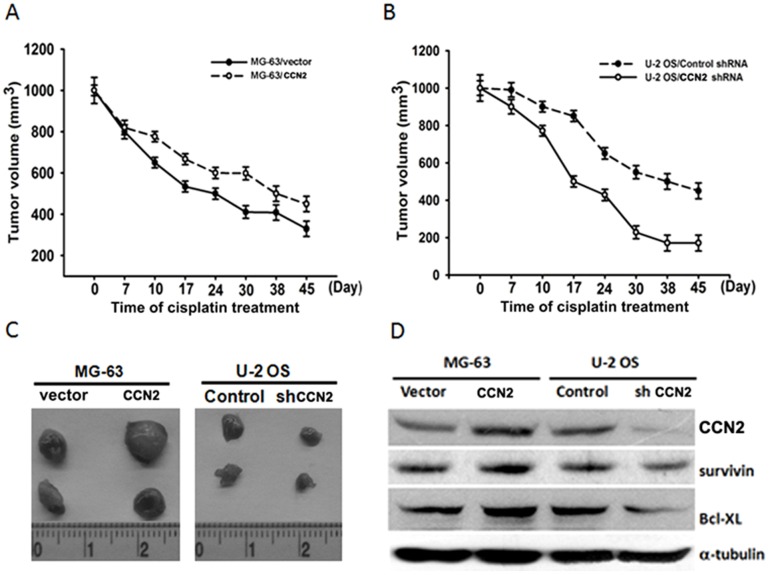
CCN2 enhances resistance to cisplatin in a mouse xenograft model. (A & B) Tumor growth curves of human osteosarcoma cells, which were treated with cisplatin for 45 days. (C) Representative photographs of MG-63/vector, MG-63/CCN2, U-2 OS/control shRNA, and U-2 OS/CCN2 shRNA cells from nude mice. (D) Western blotting was used to determine the protein levels of CCN2, Bcl-xL, survivin, and α-tubulin in tumor cells. Results are expressed as the mean ± SEM.

We used a mouse xenograft model, to verify that CCN2 confers resistance to cisplatin *in vivo*. Nude mice were divided into 4 groups and inoculated with MG-63/vector, MG-63/CCN2, U-2 OS/Control shRNA, or U-2 OS/CCN2 shRNA cells, respectively. As tumors reached 100 mm^3^ in size, mice were treated with cisplatin. As shown in Figures 6A–C, overexpression of CCN2 in MG-63 cells promoted resistance to cisplatin. On the other hand, knockdown of CCN2 in U-2 OS cells increased the therapeutic effect of cisplatin. Finally, *ex vivo* analysis of tumors excised from mice showed significantly increasing CCN2, Bcl-xL, and survivin expression in the CCN2-overexpressing group compared to that of the control group, as shown by western blotting (Fig. 6D). However, knockdown of CCN2 had contrasting effects (Fig. 6D). These data provide *in vivo* evidence to support CCN2 as a potential oncogene that renders anti-chemotherapy effect of human osteosarcoma.

## Discussion

An increasing number of osteosarcoma patients develop resistance to chemotherapy drugs, with potential resistance mechanisms that include dysfunctional membrane transport, resistance to apoptosis, and the persistence of stem cell-like tumor cells [2], [32]. Here, we show that overexpressing CCN2 in human osteosarcoma cells enhanced resistance to cisplatin through inhibiting cisplatin-induced apoptosis and promoting tumor cell survival. CCN2 overexpression resulted in specific upregulation of Bcl-xL and survivin. CCN2 also activated FAK, MEK, and ERK survival signaling pathways to enhance cell survival during cisplatin treatment. Taken together, we suggest that CCN2 plays an important role in osteosarcoma progression by supporting tumor cell survival and drug resistance. To examine whether CCN2 protected chemoresistance is a general phenomenon, the prostate (PC3), lung (A549), and gastric (AGS) cancer cells were used. Overexpression of CCN2 protected cisplatin- or doxorubicin-mediated cell death in these cancer cells (Figure S3). Therefore, CCN2 protect chemoresistance is a general phenomenon in human cancer cells.

CCN2 plays important roles in many biological processes, including cell adhesion, migration, proliferation, angiogenesis, skeletal development, and tissue wound repair. CCN2 is also critically involved in several forms of cancers [11], [12], [13], although there is dispute about the role of CCN2 in tumor carcinogenesis and its association with malignancy. For example, in human rhabdomyosarcoma, CCN2 has been demonstrated to be a useful therapeutic agent and disrupting CCN2 expression using CCN2-neutralizing antibodies can enhance apoptosis and inhibit angiogenesis [33]. In human lung adenocarcinoma, CCN2 inhibits metastasis and invasion by a CRMP-1-dependent mechanism [34]. CCN2 also inhibits cell growth in squamous cell carcinoma [35]. Additionally, CCN2 presence has been shown to be a survival factor [36]. In colorectal cancer, patients showed better overall survival when tumors displayed higher CCN2 expression. Alterations to the protein level of CCN2 in colorectal cancer cell lines also negatively modulated their invasive ability [37]. In breast cancer, CCN2 expression confers resistance to chemotherapeutic agents through augmenting a survival pathway [38]. However, the effect of CCN2 in human osteosarcoma is largely unknown. In the current study, we found that cisplatin increased osteosarcoma cell death through an apoptotic mechanism, using TUNEL staining, DAPI staining, and cell cycle analysis. In addition, overexpression of CCN2 increased the resistance to cisplatin-mediated cell apoptosis. To the best of our knowledge, this study provided the first evidence that CCN2 provides enhanced chemoresistance to cisplatin in human osteosarcoma. Therefore, CCN2 may be a novel chemotherapy target in human osteosarcoma.

Most of the pro- and anti-apoptotic members of the Bcl-2 protein family have been shown to modulate the response to cisplatin. In some cancers, including head and neck cancer, ovarian cancer, breast cancer, and non-small-cell lung carcinoma (NSCLC), the Bcl-2 family of proteins correlates to cisplatin resistance and tumor recurrence [39]. Inhibition of Bcl-xL expression is essential for therapeutic apoptosis and enhanced chemosensitivity in osteosarcoma cancer cells [40]. Here, we found that CCN2 increased Bcl-xL but not Bcl-2, Bax, Bak, and Bad expression. Bcl-xL siRNA diminished CCN2-mediated chemoresistance to cisplatin. Therefore, Bcl-xL is the most important effector in CCN2-mediated chemoresistance of the Bcl-2 family. On the other hand, increased levels of survivin have been found in gastric, esophageal, ovarian cancer, and NSCLC patients [41]; deregulation of survivin expression in various cancer types leads to increased cisplatin-resistance in patients and is associated with a negative prognosis [42]. In this study, we used specific siRNAs against survivin, which significantly abolished cisplatin-induced CCN2-mediated resistance to apoptosis. We strongly believe that survivin is a critical factor for human osteosarcoma therapy. Cisplatin induces apoptosis of cancer cells, which usually raise the defects in apoptotic programs to provide resistance to apoptosis, and Bcl-xL and survivin are important mediators during this process.

Cancer treatments can fail because cancer cells enhance some chemical signals, by rapidly developing ways to interact with the supportive ECM. CCN2 can be ECM-associated through interactions with specific integrins, and the binding of CCN2 to integrins could activate intracellular pathways, such as cell adhesion, migration, and ECM protein deposition [43]. FAK is a potential signaling molecule that mediates the activation of integrin-mediated signaling. MEK and ERK are often upregulated in response to DNA-damaging chemotherapeutic agents, such as cisplatin [44]. In the current study, we found that CCN2 increased FAK, MEK, and ERK activation. However, CCN2 did not affect phosphorylation of PI3K and the Akt signaling cascade. Furthermore, FAK and MEK inhibitors reversed CCN2-mediated resistance to cisplatin. This was confirmed by the observation that FAK, MEK, and ERK mutants prevented the enhancement of chemoresistance in human osteosarcoma cells. We suggested that CCN2 increased FAK, MEK, and ERK survival signaling pathways and subsequently protected cisplatin-induced cell apoptosis in human osteosarcoma.

In conclusion, we showed that cisplatin-induced CCN2 expression in osteosarcoma cells promoted cell survival ability, which inhibited apoptosis and increased resistance from cisplatin treatment. Our *in vitro* and *in vivo* xenograft studies showed that overexpressing CCN2 significantly increased tumor cell survival, and suppression of CCN2 expression significantly increased drug sensitivity of osteosarcoma cells. Thus, we conclude that CCN2 might be a critical oncogene and believe these data support an investigation of CCN2 as a strategic target for osteosarcoma therapy.

## Supporting Information

Figure S1 **The cell proliferation rate of MG-63/vector and MG-63/CCN2 cells.** Cells were harvested in 0, 2, 4, and 8 days. The proliferation was examined by MTT assay. Each experiment was done in triplicate.
(TIF)

Figure S2 **Overexpression of CCN2 enhances resistance to doxorubicin-mediated cell death.** Cells were treated with doxorubicin for 24 h, and cell viability was analyzed by MTT assay. Each experiment was done in triplicate.
(TIF)

Figure S3 **Overexpression of CCN2 enhances resistance to cisplatin- and doxorubicin-mediated cell death.** Cells were treated with cisplatin or doxorubicin for 24 h, and cell viability was analyzed by MTT assay. Each experiment was done in triplicate.
(TIF)
