# Supplementary figures and images for: CCN2 Enhances Resistance to Cisplatin-Mediating Cell Apoptosis in Human Osteosarcoma
Source: PLoS One. 2014 Mar 17;9(3):e90159. doi: 10.1371/journal.pone.0090159 (PMC3956456; doi:10.1371/journal.pone.0090159)

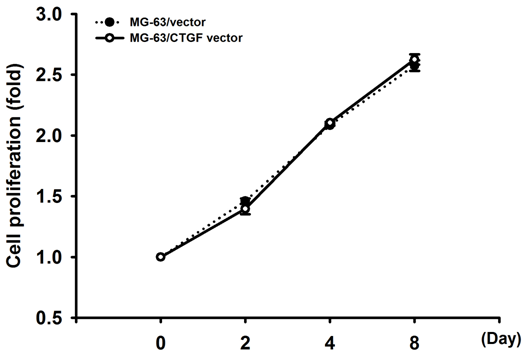

Supplement: Figure S1 — The cell proliferation rate of MG-63/vector and MG-63/CCN2 cells. Cells were harvested in 0, 2, 4, and 8 days. The proliferation was examined by MTT assay. Each experiment was done in triplicate. (TIF) [file pone.0090159.s001.tif]

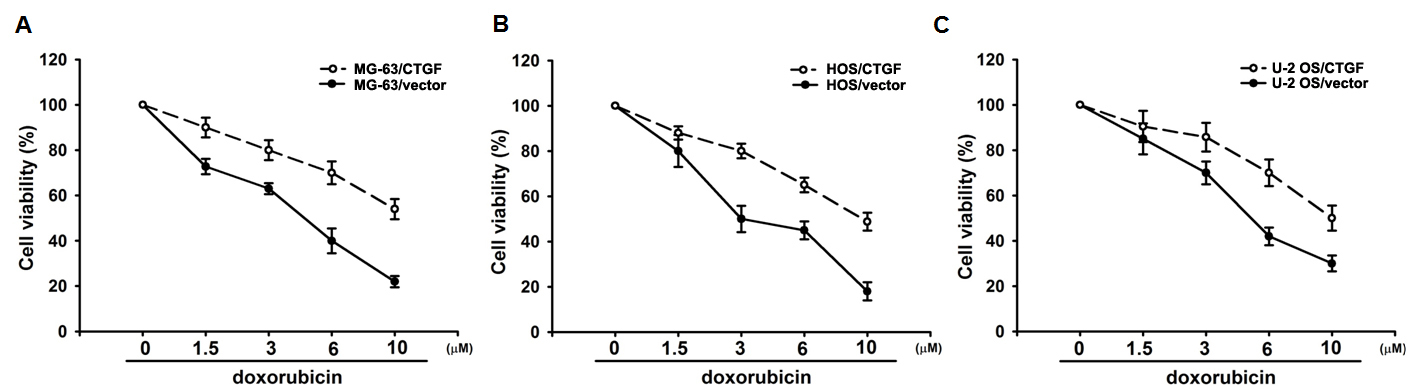

Supplement: Figure S2 — Overexpression of CCN2 enhances resistance to doxorubicin-mediated cell death. Cells were treated with doxorubicin for 24 h, and cell viability was analyzed by MTT assay. Each experiment was done in triplicate. (TIF) [file pone.0090159.s002.tif]

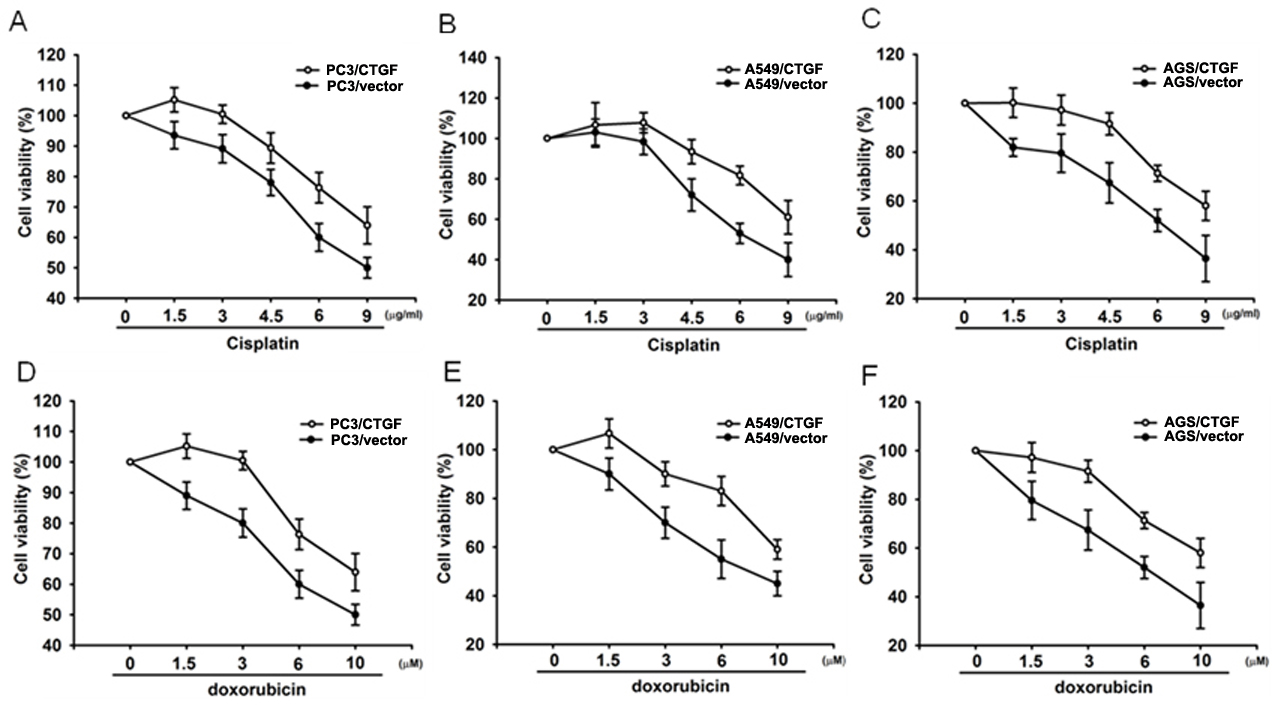

Supplement: Figure S3 — Overexpression of CCN2 enhances resistance to cisplatin- and doxorubicin-mediated cell death. Cells were treated with cisplatin or doxorubicin for 24 h, and cell viability was analyzed by MTT assay. Each experiment was done in triplicate. (TIF) [file pone.0090159.s003.tif]
